# Interleukin-1β Attenuates Myofibroblast Formation and Extracellular Matrix Production in Dermal and Lung Fibroblasts Exposed to Transforming Growth Factor-β1

**DOI:** 10.1371/journal.pone.0091559

**Published:** 2014-03-12

**Authors:** Masum M. Mia, Miriam Boersema, Ruud A. Bank

**Affiliations:** Division of Medical Biology, Department of Pathology and Medical Biology, University Medical Center Groningen, University of Groningen, Groningen, The Netherlands,; Leiden University Medical Center, Netherlands

## Abstract

One of the most potent pro-fibrotic cytokines is transforming growth factor (TGFβ). TGFβ is involved in the activation of fibroblasts into myofibroblasts, resulting in the hallmark of fibrosis: the pathological accumulation of collagen. Interleukin-1β (IL1β) can influence the severity of fibrosis, however much less is known about the direct effects on fibroblasts. Using lung and dermal fibroblasts, we have investigated the effects of IL1β, TGFβ1, and IL1β in combination with TGFβ1 on myofibroblast formation, collagen synthesis and collagen modification (including prolyl hydroxylase, lysyl hydroxylase and lysyl oxidase), and matrix metalloproteinases (MMPs). We found that IL1β alone has no obvious pro-fibrotic effect on fibroblasts. However, IL1β is able to inhibit the TGFβ1-induced myofibroblast formation as well as collagen synthesis. Glioma-associated oncogene homolog 1 (GLI1), the Hedgehog transcription factor that is involved in the transformation of fibroblasts into myofibroblasts is upregulated by TGFβ1. The addition of IL1β reduced the expression of GLI1 and thereby also indirectly inhibits myofibroblast formation. Other potentially anti-fibrotic effects of IL1β that were observed are the increased levels of MMP1, −2, −9 and −14 produced by fibroblasts exposed to TGFβ1/IL1β in comparison with fibroblasts exposed to TGFβ1 alone. In addition, IL1β decreased the TGFβ1-induced upregulation of lysyl oxidase, an enzyme involved in collagen cross-linking. Furthermore, we found that lung and dermal fibroblasts do not always behave identically towards IL1β. Suppression of COL1A1 by IL1β in the presence of TGFβ1 is more pronounced in lung fibroblasts compared to dermal fibroblasts, whereas a higher upregulation of MMP1 is seen in dermal fibroblasts. The role of IL1β in fibrosis should be reconsidered, and the differences in phenotypical properties of fibroblasts derived from different organs should be taken into account in future anti-fibrotic treatment regimes.

## Introduction

Fibrosis is the result of defective repair processes often seen after chronic injury and/or inflammation in a large variety of organs and tissues, such as the kidney, heart, liver, lung and skin. IL1β has been implicated as one of the dominant players in the development of fibrosis [1], [2], [3], [4], [5], [6], [7]. Like various organ fibrosis, lung and skin fibrosis are mediated by the IL1β [6], [7], [8]. It is expressed in the acute phase of inflammation, but is also elevated in the later stages of inflammation and tissue repair. The hallmark of fibrosis is an excessive accumulation of extracellular matrix (ECM), especially due to an imbalance between collagen synthesis and degradation [8], [9], [10], [11], [12]. One of the key processes in fibrosis is the activation of fibroblasts into myofibroblasts [13], a process that seems to be dependent on the activation of the GLI1 of the Hedgehog pathway [12], [14]. The consensus is that myofibroblasts are ultimately responsible for the excessive deposition of ECM in fibrosis.

Various cytokines play a role in the differentiation of fibroblasts into myofibroblasts. One of the major pro-fibrotic cytokines is transforming growth factor-β (such as TGFβ1), as it induces the differentiation of fibroblasts into myofibroblasts. Myofibroblasts are characterized by the presence of cytoplasmic stress fibers and show an excessive production of collagen [9], [15], [16], [17], [18], [19], [20]. TGFβ1 is also involved in the disbalance regarding the expression of matrix metalloproteinases capable of degrading collagen (e.g. MMP1, −2, −9 and −14) *versus* their inhibitors (e.g. tissue inhibitor of matrix metalloproteinase 1 and −2) [21].

As mentioned, IL1β influences the severity of fibrosis. Neutralisation or other ways of knocking down of IL1β *in vivo* results in an attenuation of fibrosis [1], [2], [3], [4],[5], whereas increasing the level of IL1β enhances the severity of fibrosis [2], [6], [7]. However, the direct effect of IL1β on fibroblasts is unclear. It has been known for a long time that fibroblasts exposed to IL1β invariably increase the expression of a variety of MMPs, and subsequently the breakdown of collagen [22], [23], [24], [25], [26], [27], [28]. This anti-fibrotic phenomenon shows a dual role for IL1β in fibrosis, as this should diminish the excessive accumulation of ECM. Conflicting data exists regarding the direct effect of IL1β on fibroblasts with respect to collagen expression/synthesis. Elevated levels of collagen expression/synthesis have been reported [29], [30], [31], but also opposite data [22], [26], [27], or no changes at all have been published [32], [33], [34]. We are not aware of studies regarding the effects of IL1β on the differentiation of fibroblasts to myofibroblasts, although a number of studies show that IL1β is able to induce epithelial to mesenchymal transition (EMT) and endothial to mesenchymal transition (EndoMT), i.e. the formation of myofibroblasts out of epithelial and endothelial cells [10], [35], [36]. Furthermore, not much is known whether IL1β and TGFβ1 have co-stimulatory effects, although both cytokines are present during tissue repair processes. Interestingly, about two decades ago it has been reported that IL1β is able to inhibit collagen type I synthesis in fibroblasts that are exposed to TGFβ [32], but this potentially anti-fibrotic finding seems to be overlooked.

In this study we investigated the effects of IL1β, TGFβ1, and a combination of IL1β and TGFβ on cultured adult human dermal and lung fibroblasts (HDFa and HLFa, respectively). The following questions were addressed: is IL1β (with or without the presence of TGFβ1) involved in (1) fibroblast to myofibroblast differentiation (including the activation of the Hedgehog pathway), (2) collagen synthesis (including the collagen-modifying enzymes prolyl hydroxylase, lysyl hydroxylase, and lysyl oxidase), and (3) increased levels of MMPs. In addition, we investigated whether fibroblasts derived from different organs (lung, skin) respond equally to pro-fibrotic stimuli. The rationale for this is that fibroblasts from internal organs show dramatic differences in gene expression patterns compared to dermal fibroblasts [37], [38].

## Materials and Methods

### Cell culture

HDFa [CCD-1093Sk (ATCC CRL-2115), ATCC, USA] and HLFa [CCD-19Lu (ATCC CCL-210), ATCC, USA] were cultured in Eagle's minimal essential medium (EMEM) (BE12-662F, Lonza Group Ltd, Switzerland) supplemented with 1% l-glutamine (Lonza, Switzerland), 1% penicillin/streptomycin (Gibco Life Technologies Ltd., UK) and 10% fetal bovine serum (FBS) (Thermo Scientific, USA) at 37°C in 5% CO_2_. Passage 7 to 12 of HDFa and HLFa were seeded with a density of 15,000 cells/cm^2^ onto a Costar 12-well plate (for quantitative real time polymerase chain reaction) or a 24-well plate (Corning Inc., USA) (for immunofluorescence staining). Cells were washed with phosphate buffer saline (PBS) after 72 h and starved for 16 h in EMEM supplemented with 1% l-glutamine, 1% penicillin/streptomycin and 0.5% FBS. Cultures were then stimulated with/without recombinant human IL1β (10 ng/ml) (201-LB-005, R&D Systems Inc, USA), recombinant human TGFβ1 (0.5, 2 and 10 ng/ml) (100–21, Peprotech, UK), or combinations thereof, for the time period as indicated. Subsequently, whole-cell lysates (as obtained with RLT-buffer; Qiagen Inc, USA), cells fixed by 1% para-formaldehyde (PFA; Merck, Germany), or collected conditioned media were used for quantitative real time polymerase chain reaction (qRT-PCR), immunofluorescence staining, and LOX activity tests, respectively.

### RNA isolation, cDNA synthesis and qRT-PCR

HDFa and HLFa were treated with IL1β and/or TGFβ1 for 24 h (or 48 h) or pre-treated with TGFβ1 (48 h) followed by IL1β stimulation for 48 h. Total RNA was isolated using the RNeasy Kit (Qiagen Inc., USA) according to the manufacturer's protocol. RNA concentration and RNA quality were measured with UV spectrophotometry (NanoDrop Technologies, Wilmington, NC). For the synthesis of cDNA, total RNA was reverse transcribed with the First Strand cDNA synthesis kit (Fermentas UAB, Lithuania) according to the manufacturer's protocol. Gene expression analysis was performed by means of qRT-PCR in a 10 μl reaction mixture containing 10 ng cDNA, SYBR Green Supermix (Bio-Rad, USA), 6 μM forward primer, and 6 μM reverse primer (for primer sequences see Table 1). qRT-CPR was performed in triplicate for each condition in a 384-well plate at 95°C for 15 sec and 60°C for 1 min for 40 cycles using the ViiA 7 Real-Time PCR System (Applied Biosystems, USA). Data was analysed with the ViiA 7 Real-Time PCR System Software v1.1 (Applied Biosystems, USA).

**Table 1:** List of primers used for qRT-PCR analysis.

Gene	Forward Sequence	Reverse Sequence
ACTA2	CTGTTCCAGCCATCCTTCAT	TCATGATGCTGTTGTAGGTGGT
COL1A1	GGGATTCCCTGGACCTAAAG	GGAACACCTCGCTCTCCA
COL3A1	CTGGACCCCAGGGTCTTC	CATCTGATCCAGGGTTTCCA
GLI1	CAGGGAGGAAAGCAGACTGA	ACTGCTGCAGGATGACTGG
GLI1ΔN	CCAGACAGAGGCCCACTC	CCCGCTTCTTGGTCAACTT
LOX	GGATACGGCACTGGCTACTT	GACGCCTGGATGTAGTAGGG
MMP1	GCTAACCTTTGATGCTATAACTACGA	TTTGTGCGCATGTAGAATCTG
MMP2	CCCCAAAACGGACAAAGAG	CTTCAGCACAAACAGGTTGC
MMP9	GAACCAATCTCACCGACAGG	GCCACCCGAGTGTAACCATA
MMP14	TACTTCCCAGGCCCCAAC	GCCACCAGGAAGATGTCATT
P4HA1	AAGATCTAACAGGACTAGATGTTTCCA	TCCTCCAACTCCATAATTTGC
P4HB	GGAATGGAGACACGGCTTC	TTCAGCCAGTTCACGATGTC
PLOD1	GAAGCTCTACCCCGGCTACT	CTTGTAGCGGACGACAAAGG
PLOD2	ATGGAAATGGACCCACCAA	TGCAGCCATTATCCTGTGTC
TAGLN	GGCCAAGGCTCTACTGTCTG	CCCTTGTTGGCCATGTCT
YWHAZ	GCAATTACTGAGAGACAACTTGACA	TGGAAGGCCGGTTAATTTT

### Immunofluorescence staining for αSMA and SM22α

HDFa and HLFa were cultured in 24-well plates and treated for 48 h with (1) IL1β, (2) TGFβ1, or (3) a combination of both. In another experiment, fibroblasts were pre-treated with TGFβ1 (48 h) followed by IL1β stimulation for 48 h. After treatment, cells were washed with PBS and fixed with 1% PFA in PBS for 15 min at room temperature (RT). Cells were then permeabilized with 0.5% Triton X-100 (Merck, Germany) in PBS for 3 min at RT. Subsequently, cells were washed and incubated for 1 h at RT with (1) monoclonal mouse anti-human to αSMA (M0851, Dako, Denmark) or (2) polyclonal rabbit to SM22α (ab14106, Abcam, UK); both primary antibodies were diluted 1∶100 in PBS containing 2% bovine serum albumin (BSA) (K1106, Sanquin reagents, Netherlands). After washing with PBS, cells were incubated for 30 min at RT with biotinylated secondary antibody (1) goat-anti-mouse (1080-08, SouthernBiotech, USA) (1∶100) or (2) goat anti-rabbit (E0432, Dako, Denmark) (1∶100) diluted in PBS containing 2% BSA for 30 min at RT. The cells were washed again and incubated with streptavidine-CY3 (Invitrogen, USA) (1∶100) in PBS containing 1% BSA and DAPI (1∶10,000) for 30 min. After washing with PBS, cell culture wells were mounted with citifluor (Agar Scientific, UK) and analyzed by fluorescence imaging microscopy (TissueFAXS, TissueGnostics GmbH, Austria), which is a valid method of quantification of immunofluorescent staining. Briefly, immunofluorescence staining was performed in a 24-well culture plate. The total area of the well was scanned by the TissueFAXS after which we used the TissueQuest software to quantify the total number of nuclei and the number of nuclei positive for the protein visualized in that particular experiment. The TissueQuest quantification results in scattergram. Each dot in a scattergram represent a nucleus and the fluorescent intensity as a measure for the amount of protein present of that particular cell can be determined on the Y-axis. Data are presented as a percentage of positive cells (mean ± SEM) of four to six independent experiments.

### Collagen type I staining

HDFa and HLFa were treated for 7 days with (1) IL1β, (2) TGFβ1, or (3) both. Additionally, Vitamin C (Sigma-Aldrich, USA) was added to the cell culture media. For collagen type I detection, we followed the same protocol as described above to detect αSMA and SM22α. To observe the production of intracellular and extracellular collagen, stainings were performed with or without Triton x-100 (Merck, Germany) permeabilization. Monoclonal mouse to collagen type I (1∶100) (ab90395, Abcam, UK) was used as primary antibody, and a biotinylated goat-anti-mouse (1∶100) as secondary antibody (SouthernBiotech, USA). Collagen type I immunofluorescence was visualized using TissueFAXS as described above.

### LOX activity assay

To measure the activity of LOX, conditioned media of fibroblasts treated for 24 and 48 h with (1) IL1β, (2) TGFβ1, or (3) both were used. LOX activity was determined with the Amplite Fluorimetric Lysyl Oxidase Assay Kit (AAT Bioquest Inc, USA) in accordance to the manufacturer's protocol. Briefly, 50 μl of assay reaction mixture was prepared, followed by the addition of 50 μl of conditioned media. The mixture was incubated at 37°C for 20 min; fluorescence intensity was detected at Excitation/Emission 540/590 nm with a Varioskan Flash Multimode Reader. Background fluorescence was subtracted and the conditioned media values were calculated and expressed as relative fluorescence intensity (RFU). To present the LOX activity, value of each treatment were plotted as bar graph. Data are presented as mean ± SEM for two independent experiments. The whole reaction was performed in a black 96-well plate.

### Statistics

All mRNA data were normalized against the reference gene tyrosine 3-monooxygenase/tryptophan 5-monooxygenase activation protein, zeta polypeptide (YWHAZ). Data are presented as mean ± SEM for at least three independent experiments. All immunofluorescence data are presented as mean ± SEM for at least four independent experiments. Results were analysed either with a one-way analysis of variance (ANOVA) followed by Tukey's post-test analysis or two-way ANOVA followed by Bonferroni post-test analysis using Graph-Pad Prism Version 5 (GraphPad Software Inc., USA). *P*<0.05 was considered to be statistically significant. *** *P*<0.001, ** *P*<0.01, * *P*<0.05.

## Results

### Dose-dependent effect of cytokines

**Figure 1:**
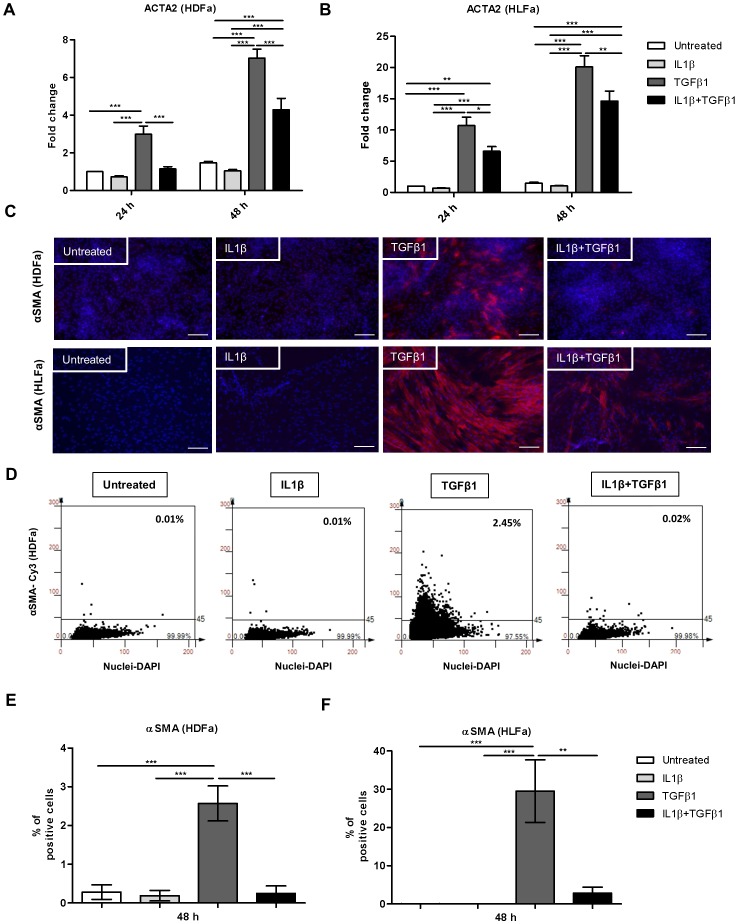
Effects of IL1β on TGFβ1-induced αSMA synthesis in dermal and lung fibroblasts. HDFa and HLFa were treated with IL1β, TGFβ1, or a combination of both, for 24 and 48 h. (A–B) The mRNA levels of ACTA2 of HDFa and HLFa were measured with qRT-PCR and expressed as fold change compared to untreated control. (C) Representative immunofluorescence stainings for αSMA are shown for HDFa (upper panel) and HLFa (lower panel). White scale bar represents 100 μm. (D) Representative TissueFAXS plots showing the % of positive cells (upper right corner) for αSMA positive fibroblasts in HDFa. (E–F) Quantification of the % of cells positive for αSMA are shown for HDFa and HLFa respectively.

To investigate the optimal concentration of TGFβ1, we performed a dose-dependent titration experiment with three concentrations of TGFβ1 (0.5, 2 and 10 ng/ml) in combination with 10 ng/ml of IL1β for 48 hours on both dermal and lung fibroblasts. Gene expression analysis showed that stimulation of fibroblasts with 0.5 ng of TGFβ1 did not increase the expression of ACTA2 in HDFa (Fig. S1A). However, 0.5 ng of TGFβ1 was sufficient to significantly increase the expression of ACTA2 in HLFa (Fig. S1B). A higher dose of TGFβ1 (2 ng/ml) did increase the expression of ACTA2 in both types of fibroblasts (Fig. S1A–B), as well as that of other genes (GLI1, COL1A1, PLOD2) that are associated with the differentiation of fibroblasts into myofibroblasts (Fig. S1C–F and S2A–B). However, both in HDFa and HLFa, the increase in gene expression levels as a result of stimulation with 2 ng/ml of TGFβ1 often did not reach the levels of the experiments with 10 ng/ml TGFβ1 (for example Fig. 1A–B).

In the titration experiment, IL1β did not reduce the expression of ACTA2 in HLFa (Fig. S1B) and GLI1 in HDFa (Fig. S1C) when fibroblasts were treated with either 0.5 or 2 ng/ml of TGFβ1, although this was seen for 10 ng/ml (for example Fig. 1A–B). However, IL1β was able to downregulate the TGFβ1-induced COL1A1 gene expression on both types of fibroblast (Fig. S1E–F). We did find a dose-dependent effect of TGFβ1 on the gene expression levels of MMP1 (Fig. S2C–D).

These findings suggest that a lower dose of TGFβ1 was sufficient to increase the gene expression of several genes associated with myofibroblast differentiation and matrix remodelling. However, the increase in gene expression after stimulation with 0.5 and 2 ng/ml of TGFβ1 was quite limited for some genes. Therefore, throughout this paper, we describe the findings of the effects of 10 ng/ml TGFβ1 and IL1β.

### IL1β inhibits TGFβ1-induced αSMA synthesis

**Figure 2:**
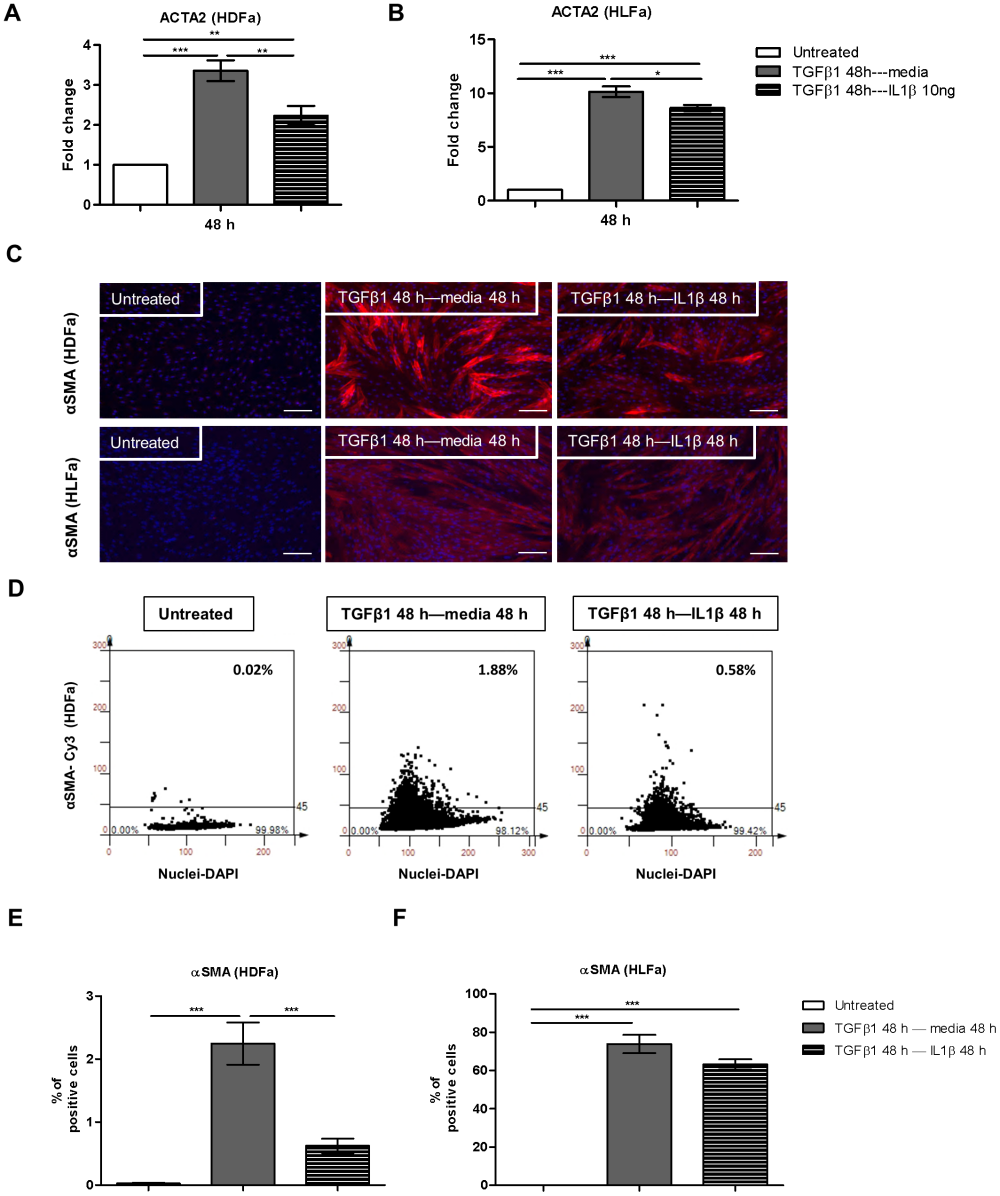
Post-treatment effects of IL1β on TGFβ1-induced αSMA synthesis in dermal and lung fibroblasts. Fibroblasts were pre-treated with TGFβ1 for 48 h followed by IL1β for 48 h. (A–B) The mRNA levels of ACTA2 of HDFa and HLFa were measured with qRT-PCR and expressed as fold change compared to untreated control. (C) Representative immunofluorescence stainings for αSMA are shown for HDFa (upper panel) and HLFa (lower panel). White scale bar represents 100 μm. (D) Representative TissueFAXS plots showing the % of positive cells (upper right corner) for αSMA positive fibroblasts in HDFa. (E–F) Quantification of the % of cells positive for αSMA are shown for HDFa and HLFa respectively.

TGFβ1 is the principal cytokine involved in the transition of fibroblasts into myofibroblasts [15], [39]. Myofibroblasts show, in contrast to fibroblasts, extensive cytoplasmic stress fibers with alpha smooth muscle actin (αSMA) and smooth muscle protein 22-alpha (SM22α) as its components [40], [41], [42]. In order to examine the effect of IL1β on TGFβ1-induced αSMA expression, fibroblasts were treated either with IL1β, TGFβ1, or both for 24 or 48 h (Fig. 1A–F). In addition, cells were pre-treated with TGFβ1 for 48 h followed by incubation with IL1β for 48 h (Fig. 2A–F). The results showed that IL1β alone did not have an effect on smooth muscle actin alpha 2 (ACTA2) mRNA expression compared to untreated cells (Fig. 1A–B). However, ACTA2 expression was significantly increased in TGFβ1-stimulated cells over time, and ACTA2 gene expression was significantly decreased when the two cytokines were combined in HDFa and HLFa at both time points (Fig. 1A–B). Even post-treatment with IL1β significantly decreased the TGFβ1-induced ACTA2 gene expression (Fig. 2A–B). To confirm the mRNA expression data on protein level, changes in cytoskeleton structure of fibroblasts and myofibroblasts were investigated by immunofluorescence staining for αSMA. In line with the mRNA data, IL1β alone had no effect on actin stress fiber formation in quiescent HDFa and HLFa, and stimulation with TGFβ1 resulted in the formation of actin stress fibers in both cell types (Fig. 1C–F). Furthermore, in a TGFβ1-rich environment, IL1β counteracted the TGFβ1-induced actin stress fiber formation, both in a combined (Fig. 1C–F) and in a post-treatment fashion in both HLFa and HDFa (Fig. 2C–F). Thus, from the αSMA data we conclude that IL1β is able to diminish TGFβ1-induced myofibroblast formation.

### IL1β inhibits TGFβ1-induced SM22α synthesis

**Figure 3:**
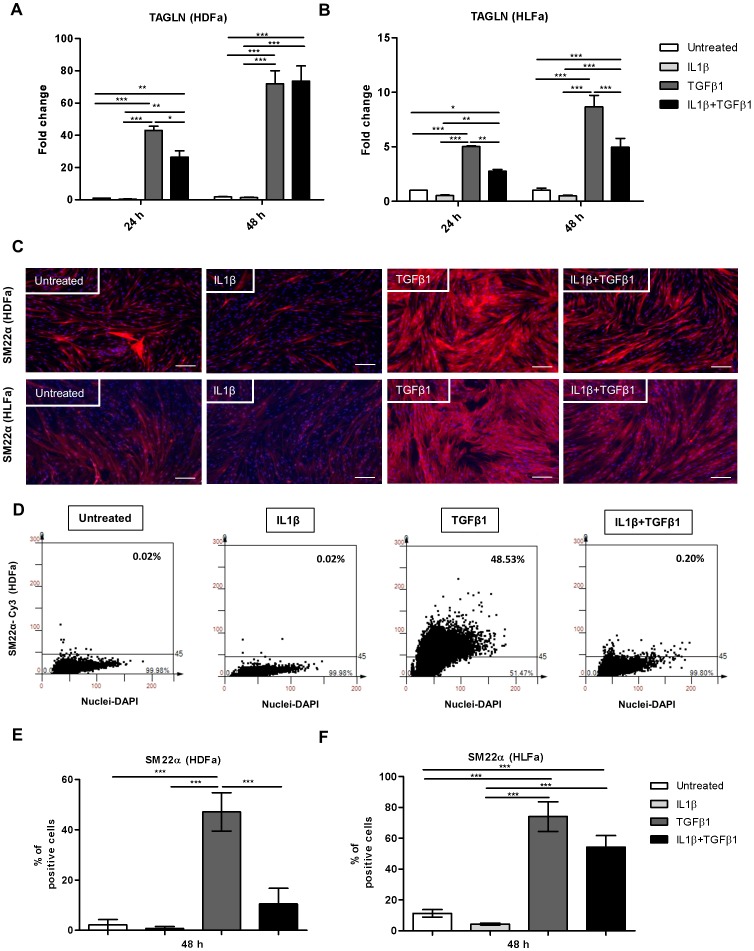
Effects of IL1β on TGFβ1-induced SM22α synthesis in dermal and lung fibroblasts. HDFa and HLFa were treated with IL1β, TGFβ1, or a combination of both, for 24 and 48 h. (A–B) The mRNA levels of TAGLN of HDFa and HLFa were measured with qRT-PCR and expressed as fold change compared to untreated control. (C) Representative immunofluorescence stainings for SM22α are shown for HDFa (upper panel) and HLFa (lower panel). White scale bar represents 100 μm. (D) Representative TissueFAXS plots showing the % of positive cells (upper right corner) for SM22α positive fibroblasts in HDFa. (E–F) Quantification of the % of cells positive for SM22α are shown for HDFa and HLFa respectively.

**Figure 4:**
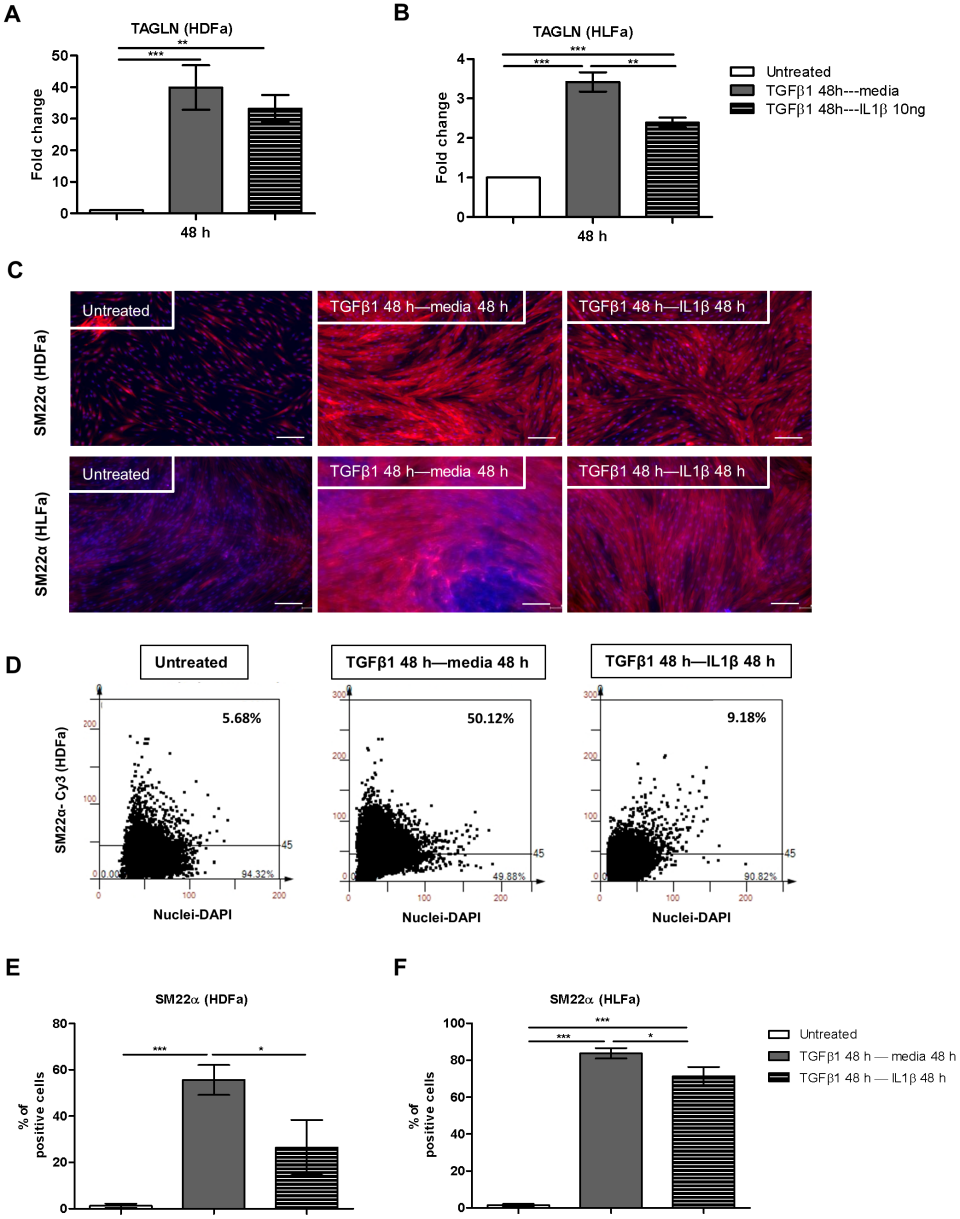
Post-treatment effects of IL1β on TGFβ1-induced SM22α synthesis in dermal and lung fibroblasts. Fibroblasts were pre-treated with TGFβ1 for 48 h followed by IL1β for 48 h. (A–B) The mRNA levels of TAGLN of HDFa and HLFa were measured with qRT-PCR and expressed as fold change compared to untreated control. (C) Representative immunofluorescence stainings for SM22α are shown for HDFa (upper panel) and HLFa (lower panel). White scale bar represents 100 μm. (D) Representative TissueFAXS plots showing the % of positive cells (upper right corner) for SM22α positive fibroblasts in HDFa. (E–F) Quantification of the % of cells positive for SM22α are shown for HDFa and HLFa respectively.

Although αSMA is the most prominent marker of myofibroblasts, these cells are also characterized by elevated levels of SM22α, which is an actin isoform. We therefore evaluated whether IL1β has similar effects on SM22α. Fibroblasts treated with IL1β already showed a trend of decreasing transgelin (TAGLN) mRNA levels in quiescent HLFa (Fig. 3B). As expected, an upregulation of TAGLN was seen in TGFβ1-treated cells. As observed with ACTA2, treatment with a combination of IL1β and TGFβ1 caused a significant downregulation of TAGLN gene expression in HDFa after 24 h and in HLFa after 24 h or 48 h (Fig. 3A–B), although no downregulation was seen for HDFa at 48 h. Immunofluorescence studies showed that untreated fibroblasts are SM22α positive, however upon IL1β stimulation the intensity of SM22α staining decreased (Fig. 3C–F). TGFβ1 stimulation highly increased the SM22α staining in HDFa and HLFa, which was significantly inhibited in HDFa in the presence of IL1β (Fig. 3C–F). Post-treatment with IL1β for 48 h significantly reduced the TGFβ1-induced upregulation of TAGLN mRNA expression in HLFa (Fig. 4B), but not in HDFa (Fig. 4A). The protein analysis revealed that post-treatment with IL1β reversed the TGFβ1-induced SM22α protein levels both in HDFa and HLFa (Fig. 4C–F). Thus, also the SM22α data shows that IL1β is able to diminish the TGFβ1-induced myofibroblast formation.

### IL1β reduces TGFβ1-induced GLI1 and GLI1ΔN gene expression

**Figure 5:**
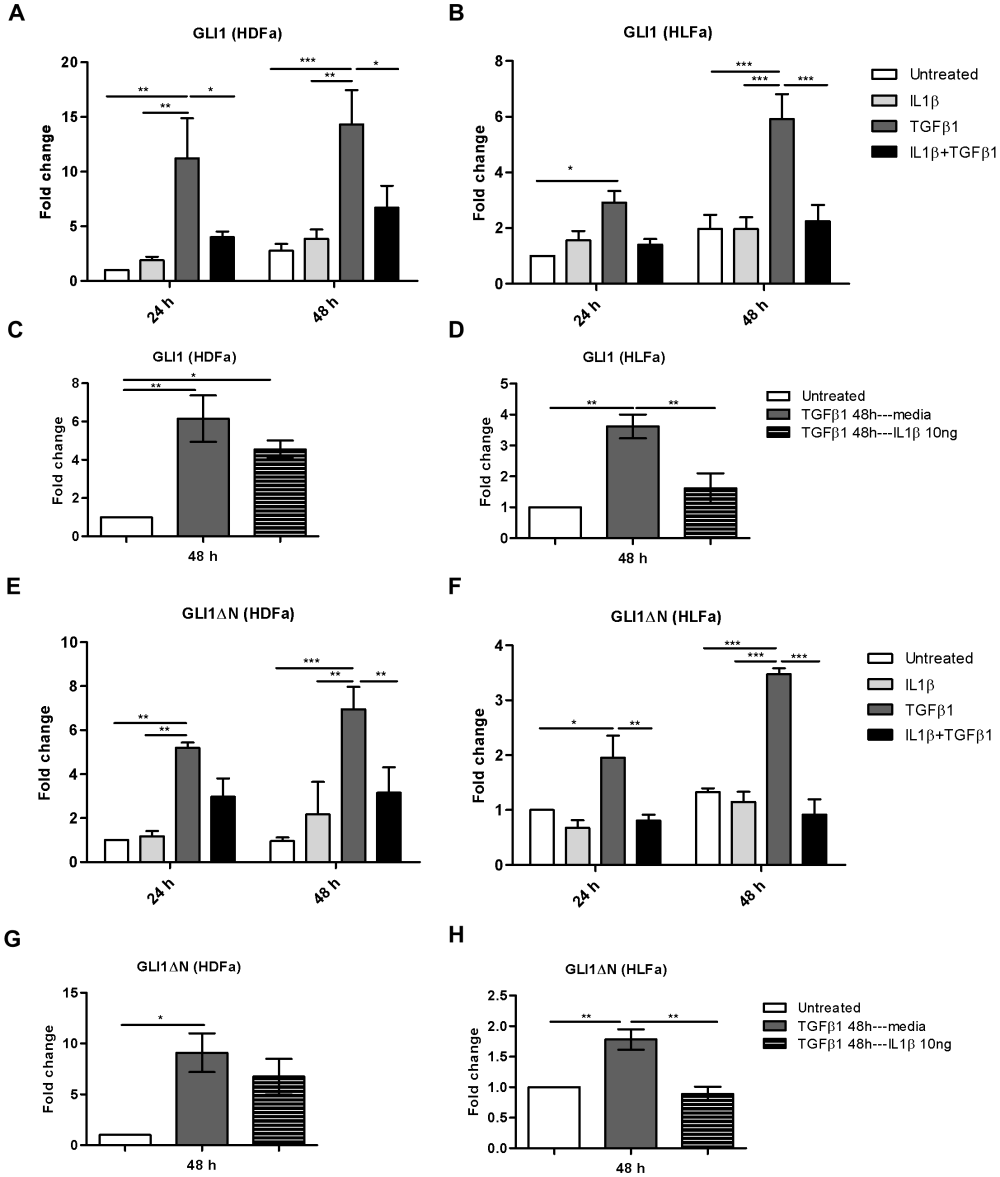
Effects of IL1β on TGFβ1-induced gene expression of Hedgehog pathway effector GLI1 and its isoform GLI1ΔN in dermal and lung fibroblasts. (A–B) HDFa and HLFa were treated with IL1β, TGFβ, or a combination of both for 24 and 48 h. The mRNA levels of GLI1 were measured with qRT-PCR and expressed as fold change compared to untreated control. (C–D) The mRNA level of GLI1 in HDFa and HLFa. Fibroblasts were pre-treated with TGFβ1 for 48 h followed by IL1β for 48 h and quantified by qRT-PCR and expressed as fold change compared to untreated control. (E–F) HDFa and HLFa were treated with IL1β, TGFβ1, or a combination of both, for 24 and 48 h. The mRNA levels of GLI1ΔN were measured with qRT-PCR and expressed as fold change compared to untreated control. (G–H) The mRNA level of GLI1ΔN in HDFa and HLFa. Fibroblasts were pre-treated with TGFβ1 for 48 h followed by IL1β for 48 h and quantified by qRT-PCR and expressed as fold change compared to untreated control.

Given the fact that GLI1 is required for the differentiation of fibroblasts into myofibroblasts [12], [14], [43], we tested whether the attenuation by IL1β of TGFβ1-induced activation of fibroblasts is indeed paralleled by decreased levels of GLI1. HDFa and HLFa were exposed to IL1β, TGFβ1, or both, for 24 h and 48 h (Fig. 5A–B). No differences were detected in GLI1 mRNA levels between IL1β-stimulated and untreated fibroblasts at both time points. GLI1 mRNA levels were significantly increased (∼10-fold in HDFa and ∼3-fold in HLFa) after TGFβ1 treatment (Fig. 5A–B). Interestingly, incubation with a combination of IL1β and TGFβ1 significantly reduced the mRNA expression of GLI1 in HDFa compared to TGFβ1 alone (Fig. 5A), whereas IL1β only significantly suppressed GLI1 expression at 48 h in HLFa (Fig. 5B), although a trend was seen at 24 h. HLFa treated with TGFβ1 for 48 followed by IL1β stimulation for 48 h showed a significant reduction in expression of GLI1; while only a trend was seen for HDFa (Fig. 5C–D). Thus, inhibition of myofibroblast differentiation by IL1β is partially reflected in decreased GLI1 levels.

Recently, an isoform of GLI1, named GLI1ΔN, has been identified [44]. However, there is limited evidence about its expression and function in fibroblasts and myofibroblasts. We asked whether GLI1ΔN also respond to IL1β- and TGFβ1-treated fibroblasts. As was the case with GLI1, the GLI1ΔN mRNA level was not affected after IL1β treatment, and was significantly upregulated up to ∼6-fold in HDFa and up to ∼3-fold in HLFa after TGFβ1 stimulation (Fig. 5E–F). Treatment of fibroblasts with IL1β and TGFβ1 together significantly decreased the expression of GLI1ΔN at 48 h compared to TGFβ1 alone (Fig. 5E–F). This was also observed when fibroblasts were treated first with TGFβ1 for 48 h, and treated afterwards for 48 h with IL1β (Fig. 5G–H), although a significance was only reached for HLFa. These findings suggest that in a pro-fibrotic environment the response of GLI1ΔN to cytokine IL1β is similar to that of GLI1.

### IL1β decreases TGFβ1-induced collagen type I synthesis, while it increases the expression of COL3A1

**Figure 6:**
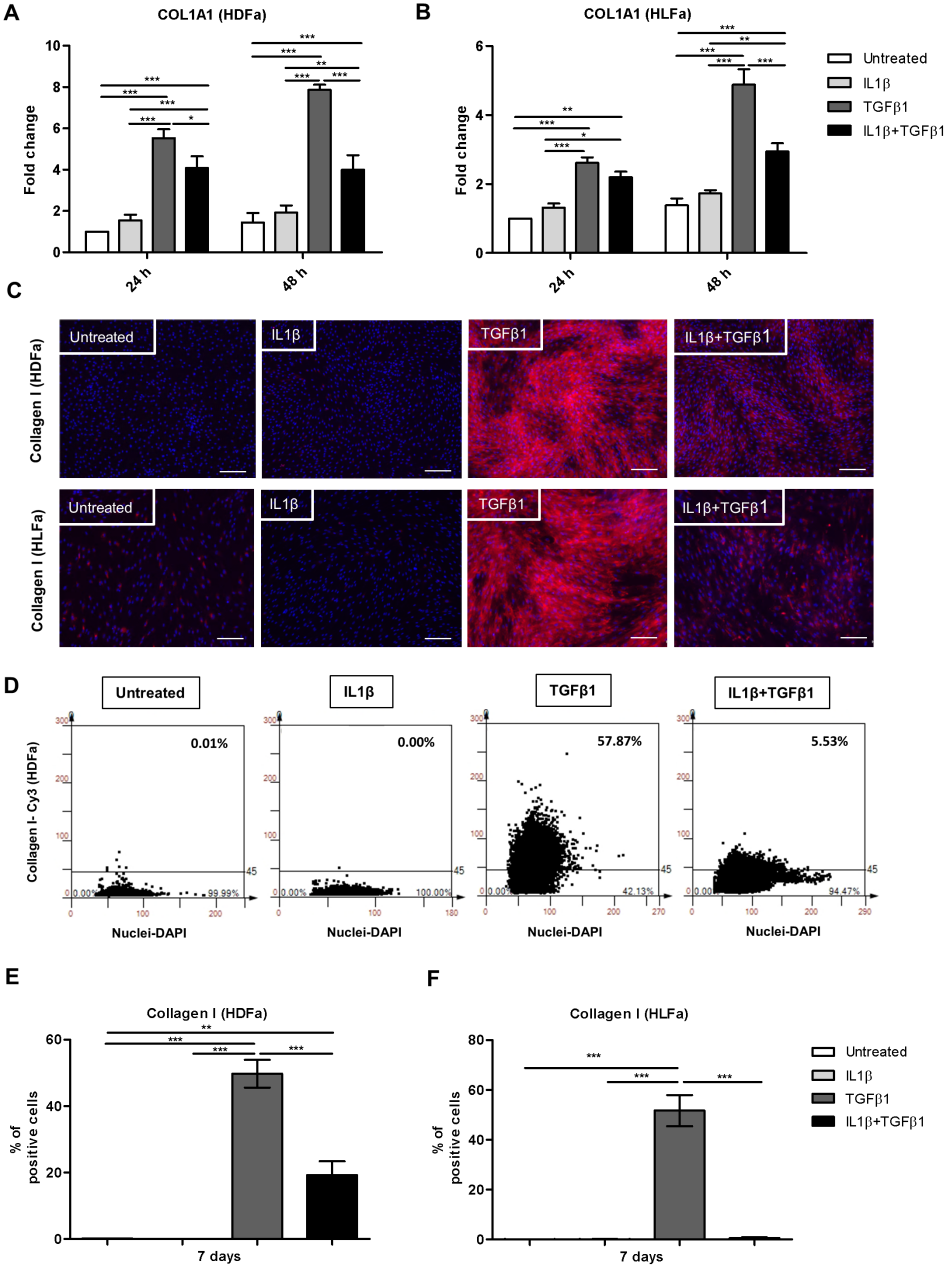
Effects of IL1β on TGFβ1-induced collagen type I synthesis in dermal and lung fibroblasts. (A–B) HDFa and HLFa were treated with IL1β, TGFβ1, or a combination of both, for 24 and 48 h. The mRNA levels of COL1A1 were measured with qRT-PCR and expressed as fold change compared to untreated control. (C) Visualisation of synthesized collagen (intracellular) by HDFa (upper panel) and HLFa (lower panel) treated for 7 days with IL1β, TGFβ1 and in combination thereof. White scale bar of each image represents 100 μm. (D) Representative TissueFAXS plots showing the % of positive cells (upper right corner) for intracellular collagen type I in HDFa. (E–F) Quantification of the % of cells positive for intracellular collagen type I are shown for HDFa and HLFa respectively.

**Figure 7:**
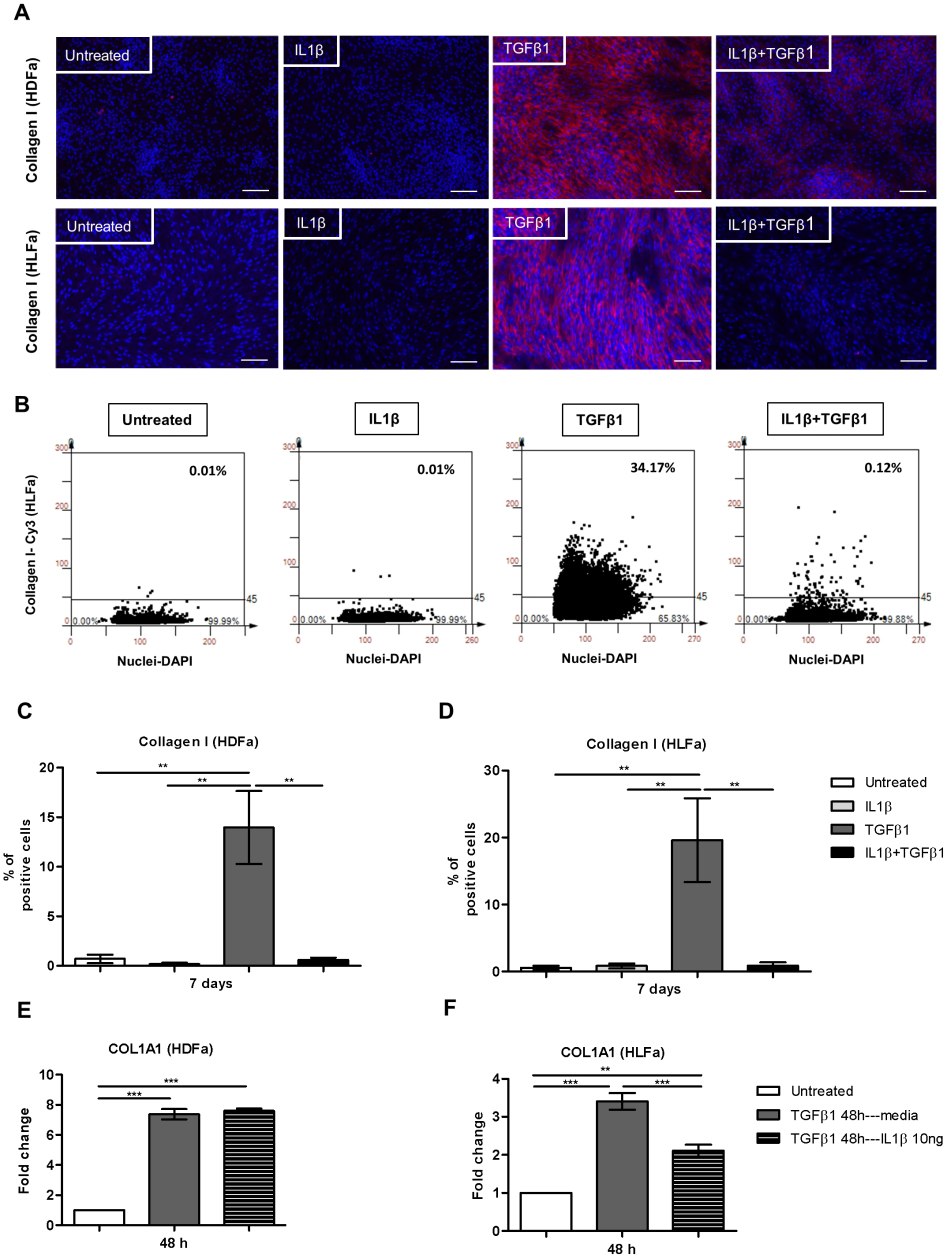
Effects of IL1β on TGFβ1-induced production of extracellular collagen type I in dermal and lung fibroblasts. (A) Visualisation of deposited extracellular collagen by HDFa (upper panel) and HLFa (lower panel) treated for 7 days with IL1β, TGFβ1 and in combination thereof. White scale bar of each image represents 100 μm. (B) Representative TissueFAXS plots showing the % of positive cells (upper right corner) for extracellular collagen type I in HDFa. (C–D) Quantification of the % of cells surrounded by extracellular collagen type I protein are shown for HDFa and HLFa respectively. (E–F) Fibroblasts were pre-treated with TGFβ1 for 48 h followed by IL1β for 48 h. The mRNA levels of COL1A1 of HDFa and HLFa were measured with qRT-PCR and expressed as fold change compared to untreated control.

Under fibrogenic conditions, myofibroblasts are responsible for the production of excessive ECM. TGFβ1 has been reported to induce accumulation of collagen type I [19], [45], [46], [47]. As we have shown above, IL1β reversed the TGFβ1-induced αSMA and SM22α expression. However, the presence of αSMA or SM22α is not required for myofibroblasts to deposit ECM. We therefore investigated whether IL1β is also able to reduce the production of ECM molecules, such as collagen, in a TGFβ-rich pro-fibrotic environment. Compared to untreated fibroblasts, IL1β did not alter mRNA levels of collagen type I alpha 1 (COL1A1), whereas TGFβ1 upregulated COL1A1 up to ∼5- to 7-fold in HDFa (Fig. 6A) and ∼2- to 4-fold in HLFa (Fig. 6B) at 24 h to 48 h, respectively. A considerable reduction of COL1A1 expression was detected at 48 h when cells were treated with a combination of TGFβ1 and IL1β (Fig. 6A–B). As expected from the mRNA data, a collagen type I staining by means of immunofluorescence revealed a strong expression both intracellular (Fig. 6C–F) and extracellular (Fig. 7A–D) in either type of fibroblasts treated with TGFβ1 alone. A considerable reduction of collagen type I staining was seen when cells were co-stimulated with IL1β (Fig. 6C and 7A). Quantification of the % of cells stained positive for intracellular collagen after 7 days demonstrated that IL1β alone had no effect, that TGFβ1 notably induced collagen synthesis, and that the combination of TGFβ1 with IL1β showed a clear trend of decreasing collagen type I synthesis in both HDFa and HLFa (Fig. 6D–F). On the other hand, quantification of the % of cells positive for extracellular deposited, collagen type I after 7 days revealed that IL1β almost completely abolished the TGFβ1-induced collagen type I production in HLFa, whereas a trend was seen for HDFa (Fig. 7A–D).

Post-treatment with IL1β also significantly decreased the mRNA level of COL1A1 in HLFa pre-treated with TGFβ1 (Fig. 7F). However, under the same conditions, no such decrease in COL1A1 mRNA levels was seen in HDFa (Fig. 7E).

**Figure 8:**
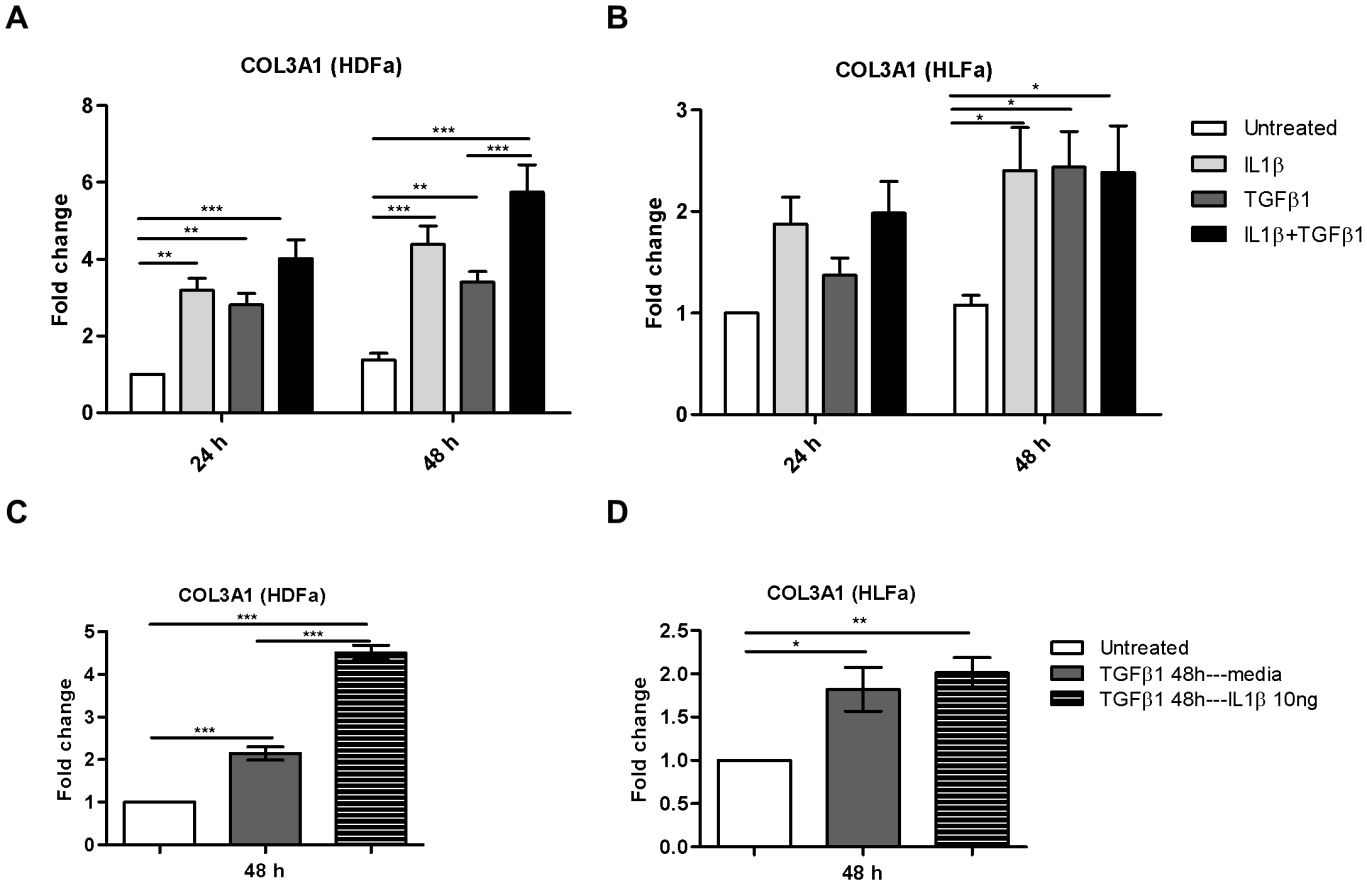
Effects of IL1β and TGFβ1 on the expression of collagen type III in dermal and lung fibroblasts. (A–B) HDFa and HLFa were treated with IL1β, TGFβ1, or a combination of both, for 24 and 48 h. The mRNA levels of COL3A1 were measured with qRT-PCR and expressed as fold change compared to untreated control. (C–D) The mRNA level of COL3A1 in HDFa and HLFa. Fibroblasts were pre-treated with TGFβ1 for 48 h followed by IL1β for 48 h and quantified by qRT-PCR and expressed as fold change compared to untreated control.

Interestingly, an opposite effect was observed for collagen type III alpha 1 (COL3A1) gene expression. Either IL1β or TGFβ1-treatment significantly increased the mRNA level of COL3A1 in HDFa at both time points (at 24 and 48 h) (Fig. 8A) and in HLFa at 48 h of incubation (Fig. 8B). The fold-increase in COL3A1 mRNA levels compared to untreated cells was higher in HDFa than in HLFa. A combination of the cytokines resulted mRNA levels of COL3A1 that were the same (in HLFa) or even higher (in HDFa) compared to fibroblasts that were treated with TGFβ1 alone (Fig. 8A–B). This was also observed when fibroblasts were treated first with TGFβ1 for 48 h, and treated afterwards for 48 h with IL1β (Fig. 8C–D). Thus, co-treatment or post-treatment with IL1β never resulted in a downregulation in mRNA expression of COL3A1, which is in contrast to what was seen with COL1A1.

### IL1β and TGFβ1 ameliorate P4HA1, P4HB, PLOD1 and PLOD2 gene expression

**Figure 9:**
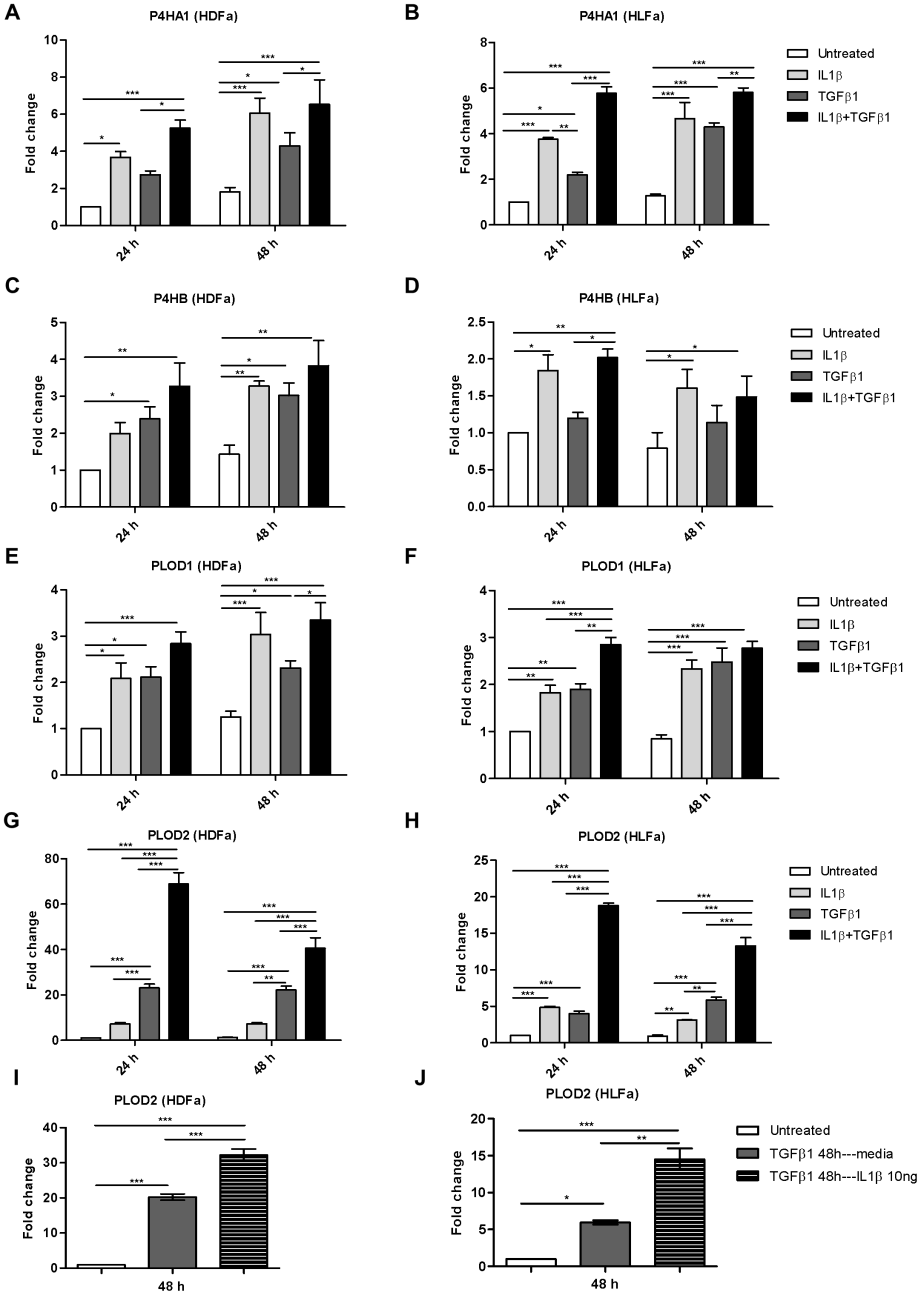
Effects of IL1β and TGFβ1 on the expression of the intracellular collagen-modifying enzymes prolyl hydroxylase (P4HA1, P4HB) and lysyl hydroxylase (PLOD1, PLOD2) in dermal and lung fibroblasts. (A–H) HDFa and HLFa were treated with IL1β, TGFβ1, or a combination of both, for 24 and 48 h. The mRNA levels of P4HA1, P4HB, PLOD1 and PLOD2 were measured with qRT-PCR and expressed as fold change compared to untreated control. (I–J) The mRNA levels of PLOD2 in HDFa and HLFa. Fibroblasts were pre-treated with TGFβ1 for 48 h followed by IL1β for 48 h and quantified by qRT-PCR and expressed as fold change compared to untreated control.

In the previous paragraph we showed that IL1β by itself does not affect COL1A1 expression, whereas TGFβ1 increases COL1A1 expression and that IL1β was able to attenuate the TGFβ1-induced expression of COL1A1. It should be noted that processing of collagen type I (as well as the other collagen types) is a complex process as several enzymes are involved in the processing and modification of collagen. Some of these enzymes play a key role in the stability of the collagen molecule/fibril. In fibrosis, an increase in the expression of collagen-modifying enzymes such as prolyl hydroxylase (e.g. P4H1, P4HB) and lysyl hydroxylase (e.g. PLOD1 and -2) has been shown in e.g. lung and skin fibrosis [48], [49], [50], [51]. TGFβ1 is able to stimulate the expression of the mentioned enzymes [52]. However, the effect of IL1β on the gene expression level of P4HA, P4HB, PLOD1 and PLOD2 is unknown. We investigated whether IL1β is able to counteract the TGFβ1-induced overexpression of these enzymes, as we have observed for COL1A1. Gene expression analysis (Fig. 9A–H) demonstrate that IL1β, like TGFβ1, significantly upregulates the expression of these enzymes in quiescent fibroblasts both at 48 h, and often also at 24 h (Fig. 9A–H). Treatment with a combination of IL1β and TGFβ1 did not result in a decrease in expression of the enzymes (which is in contrast with COL1A1); in fact, mRNA levels remained the same or were even higher compared to fibroblasts that were treated with TGFβ1 alone. The most notable increase in expression was observed of PLOD2: the expression of this enzyme was synergistically induced by the combination of IL1β with TGFβ1 (Fig. 9G–H). This was also observed when fibroblasts were treated first with TGFβ1 for 48 h, and treated afterwards for 48 h with IL1β (Fig. 9I–J). The enzyme telopeptide lysyl hydroxylase, which is encoded by PLOD2, specifically hydroxylates the lysine residues in the telopeptides, giving rise to the formation of hydroxyallysine-derived cross-links (such as pyridinoline) at the expense of allysine-derived cross-links. Pyridinoline cross-links are increased in fibrosis and seem to be associated with its irreversibility [53], [54].

### IL1β reduces TGFβ1-induced LOX mRNA expression and activity

**Figure 10:**
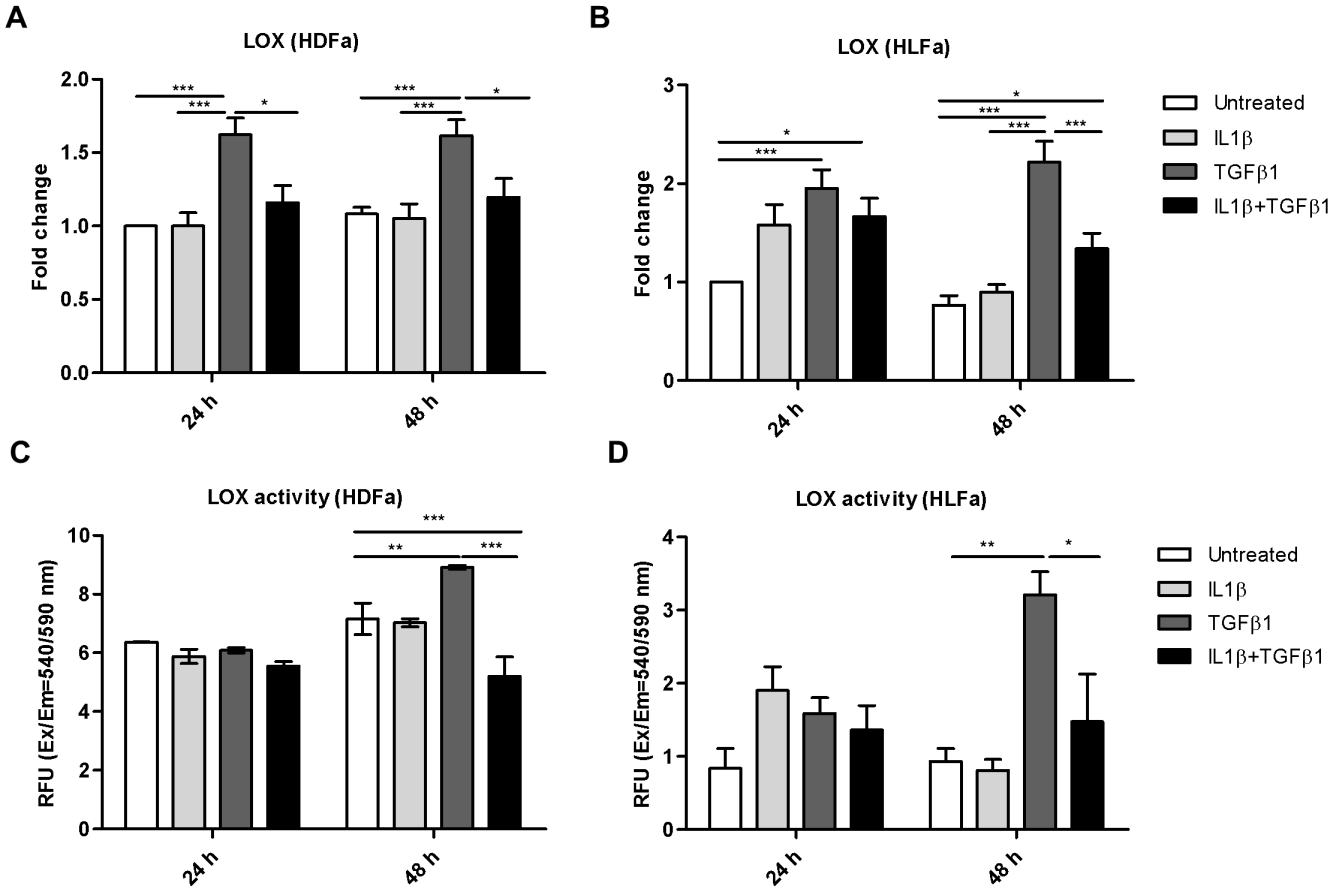
Effects of IL1β and TGFβ1 on the expression and activity level of lysyl oxidase in dermal and lung fibroblasts. (A–B) HDFa and HLFa were treated with IL1β, TGFβ1, or a combination of both, for 24 and 48 h. The mRNA levels of LOX were measured with qRT-PCR and expressed as fold change compared to untreated control. (C–D) Quantification of lysyl oxidase activity as secreted in the culture medium by HDFa and HLFa treated with IL1β, TGFβ1, or a combination of both for 24 and 48 h.

A basic step in cross-link formation is the formation of aldehydes by means of lysyl oxidase (LOX). LOX is an extracellular enzyme involved in the intermolecular cross-linking between collagen molecules within fibrils, enhancing the stability of the ECM [55]. Increased LOX activity and expression has been found in e.g. skin and lung fibrosis [56], [57], [58]. As expected, the expression was significantly increased in TGFβ1-stimulated HDFa and HLFa (Fig. 10A–B). Interestingly, the presence of IL1β resulted in a significant down-regulation of the TGFβ1-induced expression of LOX in either type of fibroblasts at 48 h (Fig. 10A–B). Fluorometric determination of LOX activity in the culture medium showed a positive correlation with mRNA data (Fig. 10C–D). Upon stimulation with IL1β, no obvious change in LOX activity was seen for HDFa and HLFa, whereas activity was increased in TGFβ1-exposed fibroblasts after 48 h. A combination of IL1β with TGFβ1 decreased the LOX activity in the culture medium of HDFa and HLFa at 48 h (Fig. 10C–D).

### IL1β activates expression of the mRNA of MMPs in TGFβ1-enriched fibroblasts

**Figure 11:**
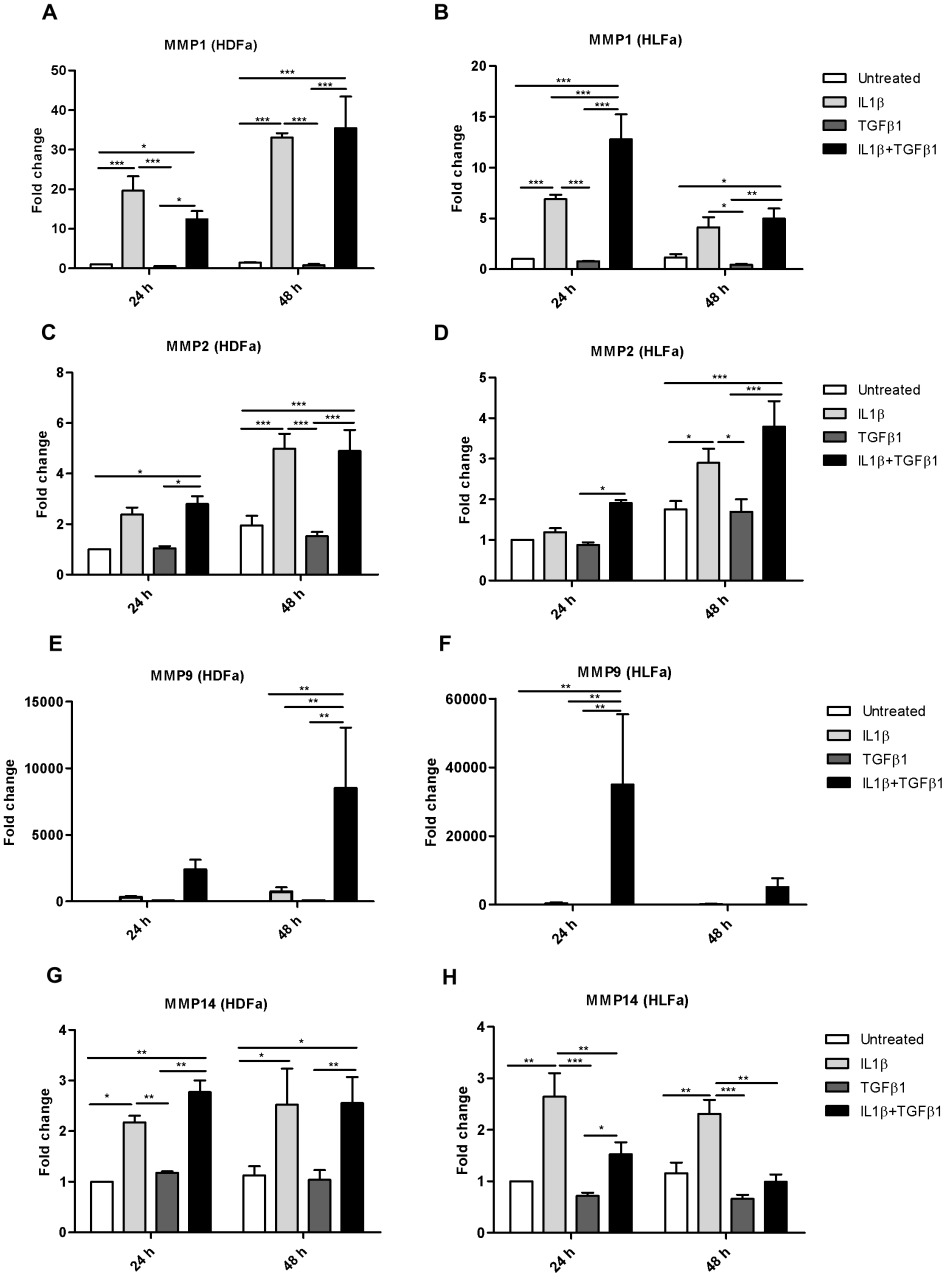
Effects of IL1β and TGFβ1 on the expression level of matrix metalloproteinases in dermal and lung fibroblasts. HDFa and HLFa were treated with IL1β, TGFβ1, or a combination of both, for 24 and 48 h. (A–H) The mRNA levels of MMP1, −2, −9 and 14 were measured with qRT-PCR and expressed as fold change compared to untreated control.

**Figure 12:**
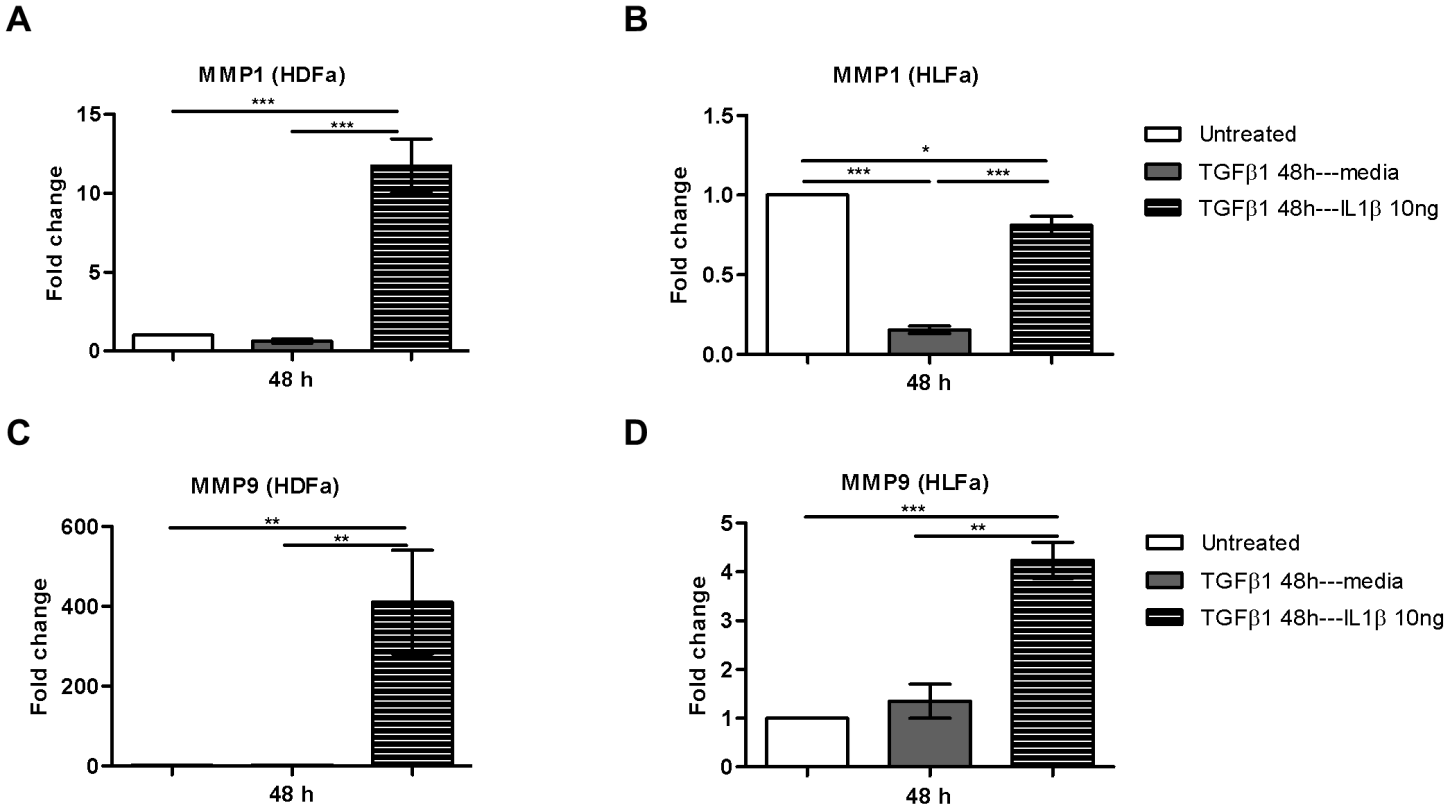
Post-treatment effects of IL1β on TGFβ1-stimulated dermal and lung fibroblasts on the expression level of matrix metalloproteinases. Fibroblasts were pre-treated with TGFβ1 for 48 h followed by IL1β for 48 h and quantified by qRT-PCR and expressed as fold change compared to untreated control. (A–B) The mRNA levels of MMP1 in HDFa and HLFa. (C–D) The mRNA levels of MMP9 in HDFa and HLFa.

In fibrosis, a disbalance is seen between collagen expression and the expression of collagenases and gelatinases: the increase in collagen expression is not accompanied with an increase in MMP expression. We investigated the effect of IL1β on the expression of MMP1, −2, −9 and −14 in a non-fibrotic (without TGFβ1) and in a pro-fibrotic (with TGFβ1) environment (Fig. 11–12). We observed that IL1β resulted in a significant upregulation of MMP1, −2 and −14 in a non-fibrotic environment both in HDFa and HLFa. As expected, TGFβ1 alone did not upregulate gene expression of the measured MMPs. Stimulation of fibroblasts with IL1β resulted in an upregulation of most MMPs compared to non-stimulated cells. The increased gene expression of MMPs after IL1β stimulation was also obvious in the presence of TGFβ1 (Fig. 11A–H and Fig. 12A–D). However, TGFβ1 did affect the level of gene expression of certain MMPs in the presence of IL1β. The increased expression of MMP14 caused by IL1β was dampened by TGFβ1 in HLFa, whereas no such attenuation was seen in HDFa (Fig. 11G–H). In contrast, the increased MMP9 levels caused by IL1β were even further upregulated in the presence of TGFβ1 (Fig. 11E–F). Fibroblasts treated with TGFβ1 for 48 h followed by IL1β treatment for 48 h showed a high upregulation of MMP9 (Fig. 12A–B). Such an upregulation was also observed for MMP1 in HDFa, whereas a pre-treatment with TGFβ1 resulted in a dampening of MMP1 expression in HLFa (Fig. 12C–D).

## Discussion

It has been reported, that IL1β has a deleterious role in fibrosis *in vivo*. However, in these studies the role of IL1β in the different phases of fibrosis, i.e. the acute inflammation phase and the later stages of inflammation and tissue repair, has not been dissected. Furthermore, relatively little is known about the direct effects of IL1β on fibroblasts, and even less is known whether IL1β influences the pro-fibrotic effect of TGFβ1 towards fibroblasts. We have investigated the direct role of IL1β on dermal and lung fibroblasts, in the presence or absence of TGFβ1, and found that IL1β alone did not contribute to the formation of myofibroblasts, and that IL1β on the contrary is able to attenuate or even reverse the pro-fibrotic effects of TGFβ1.

Although IL1β alone did show a stimulatory effect on the expression of COL3A1 and the collagen-modifying enzymes P4HA1, P4HB, PLOD1 and PLOD2, we observed that IL1β alone had no stimulatory effect of GLI1, GLI1ΔN, COL1A1, ACTA2 and TAGLN mRNA expression levels (i.e. it did not contribute to the activation of fibroblasts into myofibroblasts or to the increase in collagen type I expression). Furthermore, IL1β alone *did* have a stimulatory effect on the expression of collagenolytic enzymes MMP1, −2, −9 and −14, which is another potentially positive (i.e. anti-fibrotic) effect. A major finding of our study is further, that the co-presence of IL1β inhibited the pro-fibrotic stimulatory effects of TGFβ1, by decreasing the mRNA levels of GLI1, GLI1ΔN, COL1A1, ACTA2 and TAGLN, an effect that was verified on a protein level as well. Thus, IL1β inhibits myofibroblast formation as induced by TGFβ1, and therefore potentially could attenuate the severity of fibrosis. Other potentially positive (i.e. anti-fibrotic) effects of IL1β were the increased levels of MMP1, −2, −9 and −14 produced by fibroblasts exposed to TGFβ1/IL1β compared to fibroblasts exposed to TGFβ1 alone. In addition, IL1β decreased the TGFβ1-induced upregulation of LOX, which was verified by means of measuring total lysyl oxidase activity levels in the culture medium. Lysyl oxidase is involved in the cross-linking of collagen, making the collagen network more resistant towards proteolytic enzymes. Lowering lysyl oxidase activity levels has anti-fibrotic effects [58], [59].

The mechanisms of IL1β to attenuate the TGFβ1-induced myofibroblast formation are largely unknown. However, recent studies regarding the mechanism of fibroblast to myofibroblast transition has led to a renewed interest in the non-canonical activation of the sonic Hedgehog (SHH) pathway, especially with respect to the transcription factor GLI1 [11], [12], [14]. It has been shown that the SHH pathway is activated in fibrotic conditions [60], [61], [62], that GLI1 expression is induced by TGFβ1, and the transformation of fibroblasts into myofibroblasts is GLI1-dependent [14], [43]. In our study we also observed an upregulation of GLI1 by TGFβ1, but this was reduced by the addition of IL1β, and as a consequence myofibroblast formation was hampered. A similar effect was seen for the isoform of GLI1, GLI1ΔN. Although our study showed that regulation of GLI1ΔN was similar to GLI1, the function of GLI1ΔN needs to be further investigated. The ability of IL1β to downregulate GLI1 could potentially be anti-fibrotic, as GLI activation was found to be required for TGFβ1-induced myofibroblast differentiation [14]. Inhibition of GLI by GANT61 (i.e. directly blocking the binding of GLI1 and GLI2 to their DNA targets) abrogated the effect of TGFβ1 on protein levels of αSMA and collagen type I in human lung fibroblasts [14]. As with fibroblasts, activation of the SHH pathway results in the transdifferentiation of hepatic stellate cells into myofibroblasts, a key mechanism in liver fibrosis [63], [64], [65], [66]. Pharmacological inhibition of the SHH pathway results in an attenuation of fibrosis in a variety of *in vivo* models, namely unilateral ureteral obstruction (UUO) [67], systemic sclerosis [68] and liver cirrhosis [66], [69], [70], although others did not report improvements [71], [72].

Another potential anti-fibrotic feature of IL1β might be its ability to upregulate COL3A1. In all cases, stimulation with IL1β resulted in an increased collagen type III to collagen type I ratio, as expression of collagen type I went down and that of collagen type III went up or remained the same. Interestingly, an increased collagen III:I ratio is often found in situations where a scarless healing is observed [73]. A prerequisite is that the produced collagens are adequately modified. As stimulation with IL1β did not result in a decrease in the expression level of prolyl hydroxylase or lysyl hydroxylase, this seems to be the case.

A negative finding of our study was the steep increase in PLOD2 expression when IL1β is added to TGFβ1. Increased levels of PLOD2 are seen in fibrotic conditions [49], [50], resulting in the formation of increased levels of pyridinium cross-links *at the expense* of other cross-links, and making the collagen more difficult to degrade. However, this might be compensated with IL1β by (1) inhibiting the TGFβ1-induced upregulation of LOX (resulting in less lysyl oxidase activity levels, which likely results in a decrease in the *total* amount of cross-links), and (2) by upregulating the expression of various MMPs, which likely results in a higher collagenolytic potential.

Our data might have major implications regarding the possible use of IL1β inhibitors in fibrosis. We showed that IL1β is able to suppress the pro-fibrotic features of TGFβ1, and thus shows potential anti-fibrotic properties, at least in the later stages of tissue repair, where TGFβ levels are more prominent. It is possible that inhibition of IL1β in the early stages of tissue repair alone has better effects than continuous suppression of IL1β activity during the entire repair process. Inhibiting the early inflammatory cascade certainly has an anti-fibrotic effect, but inhibiting IL1β at the later phases of the healing response might have a negative effect on the direct anti-fibrotic properties of IL1β on fibroblasts in the presence of TGFβ. In this context it is of interest, that a single injection of an IL1β inhibitor immediately after ligation in a non-reperfused infarction model attenuated the collagen accumulation in the infarcted area [74], indicating that the timing and duration of intervention with regard to IL1β is a key determinant of outcome [75].Another relevant finding from our study is that fibroblasts from different origins (in our case lung and skin) do not always behave identical towards IL1β. Most marked in this respect is the magnitude of COL1A1 suppression by IL1β between dermal and lung fibroblasts after the pre-treatment with TGFβ1, and the magnitude of upregulation of COL3A1 and MMP1 by IL1β after the pre-treatment with TGFβ1. This difference in behaviour between dermal and lung fibroblasts is in line with the observation (albeit widely neglected) that fibroblasts derived from different organs show major differences in phenotypic properties, as reflected by huge differences in gene expression patterns [37], [38]. These phenotypical differences should be taken into account in the search for anti-fibrotic agents: an inhibitor may not have the desired effect in all organ systems due to the different response of the fibroblasts.

In conclusion we have shown that IL1β counteracts a central process in fibrogenesis, namely the TGFβ1-mediated transition of fibroblasts into myofibroblasts. Clearly, the role of IL1β in fibrosis should be reconsidered. Furthermore, we have shown that fibroblasts from different origin (i.e. lung and dermal fibroblasts) show different phenotypical responses towards IL1β. Both these findings should be taken into consideration in the development of future anti-fibrotic therapies. In the future we aim to investigate what the underlying mechanism of IL1β is to the actions of TGFβ1-induced pro-fibrotic responses in fibroblasts with respect to myofibroblast differentiation and collagen biosynthesis.

## Supporting Information

Figure S1 **Dose-dependent effects of TGFβ1 and IL1β on the genes expression level of pro-fibrotic markers in dermal and lung fibroblasts.** HDFa and HLFa were treated with TGFβ1 (0.5 and 2 ng/ml), IL1β (10 ng/ml), a combination thereof, or both, for 48 hours (A–F). The mRNA levels of ACTA2, GLI1 and COL1A1 were quantified with qRT-PCR and expressed as a fold change compare to untreated control. Gene expression data was normalized to the reference gene YWHAZ. Data are represented as mean ± SEM of quadruplicate experiments.
(TIF)

Figure S2 **Concentration-dependent effects of TGFβ1 and IL1β on the gene expression level of the collagen-modifying and -degrading enzymes in dermal and lung fibroblasts.** HDFa and HLFa were treated with TGFβ1 (0.5 and 2 ng/ml), IL1β (10 ng/ml), a combination thereof, or both, for 48 hours (A–F). The mRNA levels of PLOD2, MMP1 and MMP9 were quantified with qRT-PCR and expressed as a fold change compare to untreated control. Gene expression data was normalized to the reference gene YWHAZ. Data are represented as mean ± SEM of quadruplicate experiments.
(TIF)

Methods S1 **Cell culture and qRT-PCR.**
(DOCX)
