# Supplementary figures and images for: Interleukin-1β Attenuates Myofibroblast Formation and Extracellular Matrix Production in Dermal and Lung Fibroblasts Exposed to Transforming Growth Factor-β1
Source: PLoS One. 2014 Mar 12;9(3):e91559. doi: 10.1371/journal.pone.0091559 (PMC3951452; doi:10.1371/journal.pone.0091559)

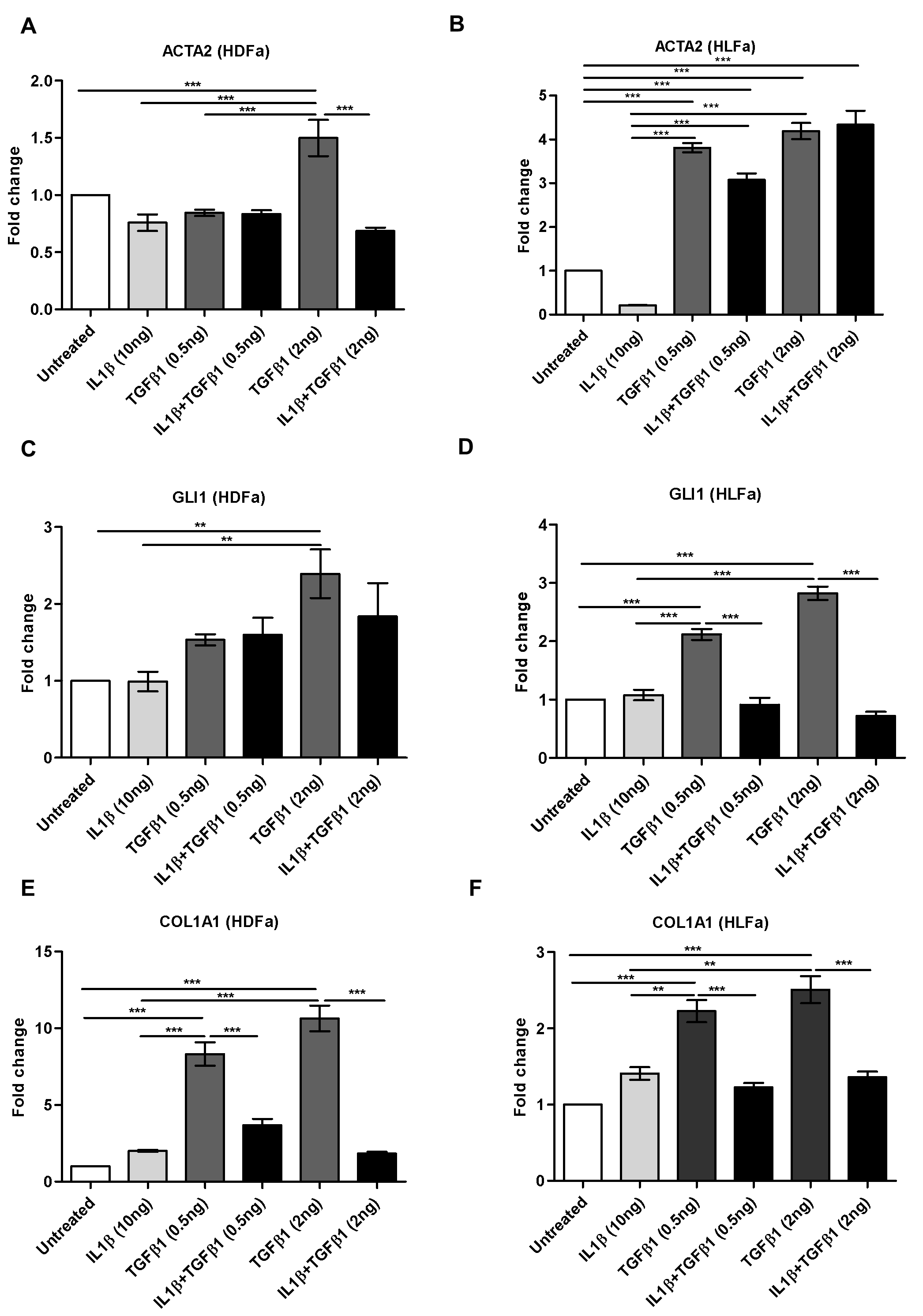

Supplement: Figure S1 — Dose-dependent effects of TGFβ1 and IL1β on the genes expression level of pro-fibrotic markers in dermal and lung fibroblasts. HDFa and HLFa were treated with TGFβ1 (0.5 and 2 ng/ml), IL1β (10 ng/ml), a combination thereof, or both, for 48 hours (A–F). The mRNA levels of ACTA2, GLI1 and COL1A1 were quantified with qRT-PCR and expressed as a fold change compare to untreated control. Gene expression data was normalized to the reference gene YWHAZ. Data are represented as mean ± SEM of quadruplicate experiments. (TIF) [file pone.0091559.s001.tif]

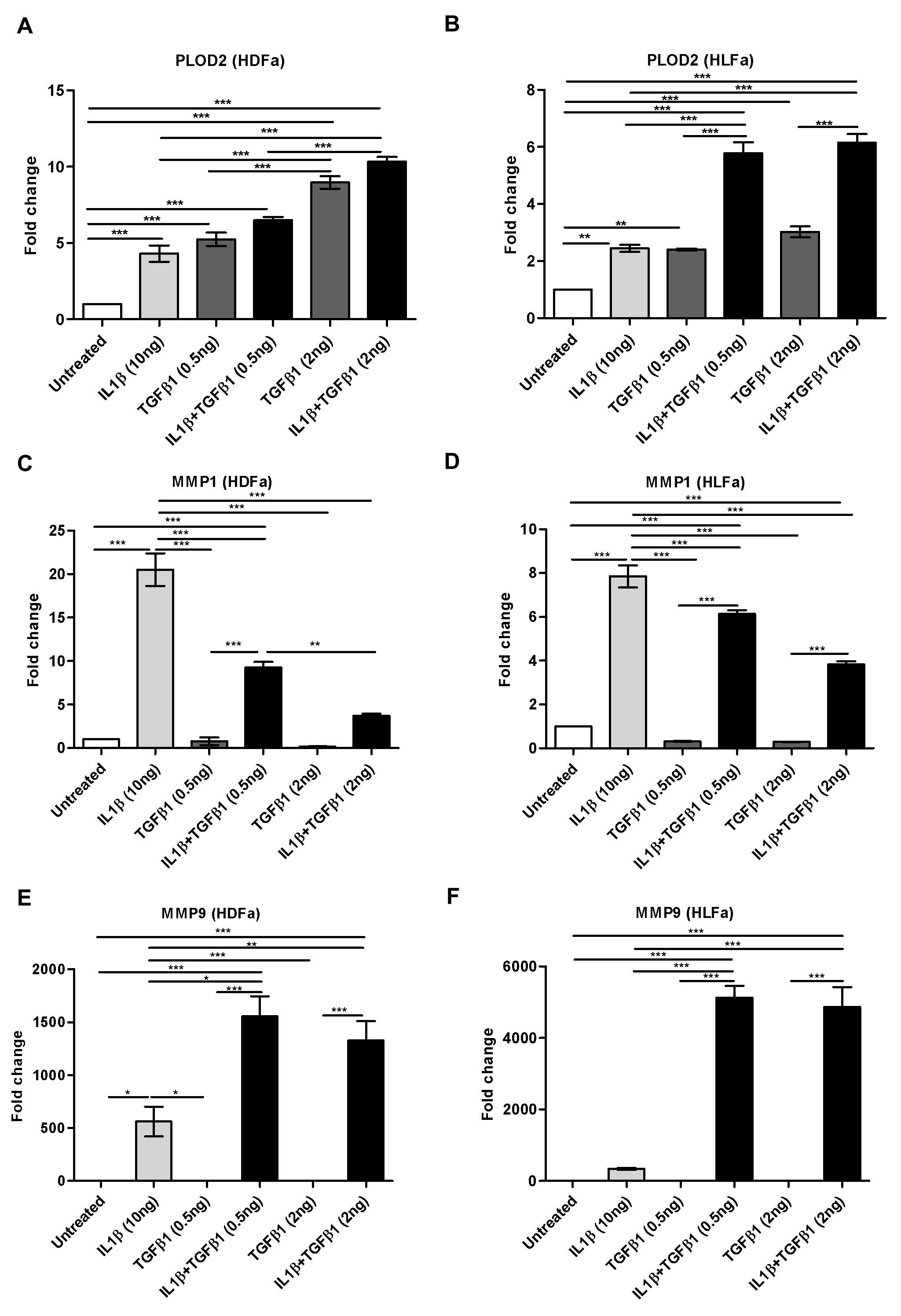

Supplement: Figure S2 — Concentration-dependent effects of TGFβ1 and IL1β on the gene expression level of the collagen-modifying and -degrading enzymes in dermal and lung fibroblasts. HDFa and HLFa were treated with TGFβ1 (0.5 and 2 ng/ml), IL1β (10 ng/ml), a combination thereof, or both, for 48 hours (A–F). The mRNA levels of PLOD2, MMP1 and MMP9 were quantified with qRT-PCR and expressed as a fold change compare to untreated control. Gene expression data was normalized to the reference gene YWHAZ. Data are represented as mean ± SEM of quadruplicate experiments. (TIF) [file pone.0091559.s002.tif]
