# Supplementary material for: Interleukin-1β Attenuates Myofibroblast Formation and Extracellular Matrix Production in Dermal and Lung Fibroblasts Exposed to Transforming Growth Factor-β1
Source: PLoS One. 2014 Mar 12;9(3):e91559. doi: 10.1371/journal.pone.0091559 (PMC3951452; doi:10.1371/journal.pone.0091559)
Supplement: Methods S1 — Cell culture and qRT-PCR. (DOCX) [file pone.0091559.s003.docx]

**Supplementary methods**

*Cell culture and qRT-PCR*

HDFa and HLFa were cultured in EMEM supplemented with 1% l-glutamine, 1% penicillin/streptomycin and 10% fetal bovine serum at 37°C in 5% CO_2_. HDFa (passage 6) and HLFa (passage 5) were seeded with a density of 15,000 cells/cm^2^ onto a Costar 12-well plate. Cells were washed with phosphate buffer saline after 72 h and starved for 16 h in EMEM supplemented with 1% l-glutamine, 1% penicillin/streptomycin and 0.5% FBS. Cultures were then stimulated with/without recombinant human IL1β (10 ng/ml), recombinant human TGFβ1 (0.5 or 2 ng/ml), or combinations thereof, for 48 hours. Subsequently, whole-cell lysates were used to isolate total RNA and to synthesize cDNA for performing quantitative real time polymerase chain reaction. Data was analysed with the ViiA 7 Real-Time PCR System Software v1.1.
